# Effect of pulsating solidification on the surface properties of conductive materials

**DOI:** 10.1098/rspa.2021.0726

**Published:** 2022-05

**Authors:** Rongshan Qin, Ashutosh Bhagurkar

**Affiliations:** School of Engineering and Innovation, The Open University, Walton Hall, Milton Keynes MK7 6AA, UK

**Keywords:** kinetics, oxide, solidification, machine learning, electrical properties

## Abstract

Application of pulsed electric current to solidifying conductive materials leads to a rapid and significant reduction of surface roughness. This is validated experimentally in the present work for multi-component oxides. The surface geometry of the casts without and with pulsating treatment was measured using a Leica DCM-3D Profiler. The pulsating treatment reduces the roughness of solidified materials by more than 50%. The unmeasurable points, which account for nearly 2% of total area, were interpolated using an artificial multi-layer neural network. Both the experimentally measured and neural network interpolated surface profile data were implemented to the calculation of electric current free energy. The results show that the electric current free energy can overtake surface energy and provide a significant driving force to the kinetics of surface evolution in solidification. Distortion of the electric field is noticed around the interface.

## 1. Introduction

Fabrication of materials to possess a desirable surface property has been a persistent challenge in manufacturing. A material fabricated with a smoother surface requires less effort in post-processing polishing to become a product, which saves energy and other costs. Recently, pulsed electric current has been found favourable in reducing surface roughness of engineering alloys. Ye *et al.* [1] reported that electropulsing-assisted ultrasonic surface rolling could reduce the surface roughness of pure titanium and Ti-6Al-4 V alloys. Akram *et al.* [2] reported that alternating magnetic field treatment, which inducts an eddy current in the materials, caused surface roughness modification for nickel-aluminium bronze and aluminium alloy. Both mentioned works used electric current in solid-state alloys. Based on thermodynamic analysis and Boltzmann distribution theory, Qin developed a theory and suggested the application of electric current pulses in solidification to modify the surface geometry [3]. This requires less energy in comparison with that in solid-state processing and is also suitable for brittle materials such as conductive ceramics. The experimental validation, however, is overdue.

From a kinetic perspective, the profile of a free surface in casts is determined by the surface fluctuation and dissipation during solidification. Fluctuation is driven by fluid convection, stress evolution, phase transition, environmental perturbation and temperature. Dissipation is controlled by several kinematic factors including viscosity and fluidity. Prof. Long reviewed the structural relaxation in materials and suggested describing it using effective structural relaxation time [4]

1.1 $$\tau =\tau_{0}\;\text{exp}\left(\frac{W}{kT}\right)\times \text{exp}\left(\frac{\Delta }{2kT}\right),$$

where *W* is the kinetic barrier. Δ is the free energy asymmetry before and after the structural relaxation. For a system with free energy density before relaxation at *g_b_* and after relaxation at *g_a_*, the asymmetry is given by Δ = *g_a_* − *g_b_*. *τ*_0_ is the intrinsic relaxation time in relation to atoms vibration. *k* is Boltzmann constant and *T* is temperature. A longer relaxation time demands a slower solidification rate in order to allow the fluctuation to dissipate to its equilibrium geometry. Δ consists of the surface energy difference and bulk free energy difference between the initial and final states. The surface energy drives surface area to minimize for those materials having positive surface tension and to maximize for the materials with negative surface tension. Most metals and alloys have positive surface tension. Some surfactants and micelles have negative surface tension. The fundamental understanding of the negative surface tension has been discussed by Mathur *et al.* [5].

The bulk free energy consists of chemical free energy and external field free energy. The strain-stress energy in liquid is negligible due to high fluidity. The chemical free energy remains constant in surface evolution due to the unchanged constitution of phases and compositions. Application of electromagnetic field to the materials changes their external field free energy. The electromagnetic free energy is described by Landau theory, which can be represented in the following equation according to Eerenstein *et al.* [6]

1.2 $$G_{e}=-\left[\frac{1}{2}\varepsilon_{0}\varepsilon_{ij}E_{i}E_{j}+\frac{1}{2}\mu_{0}\mu_{ij}H_{i}H_{j}+\alpha_{ij}E_{i}H_{j}+\frac{\beta_{ijk}}{2}E_{i}H_{j}H_{k}+\frac{\gamma_{ijk}}{2}H_{i}E_{j}E_{k}+\cdots \right],$$

where *E_i_* and *H_i_* are *i*-th component of electric and magnetic fields, respectively (e.g. in Cartesian coordinates *E_i_* contains *E_x_*, *E_y_* and *E_z_* components). The coefficients are electric permittivity (*ϵ*), magnetic permeability (*μ*) and magnetoelectric-coupling coefficients (*α*, *β* and *γ*). The first term is the electric field free energy to describe the dielectric materials. The second term is the magnetic field free energy or alternately called electric current free energy to describe the conductive materials. The third to fifth terms reflect the magnetic field-influenced electric properties and the electric field-influenced magnetic properties, which are applicable to the multi-ferroic and magnetoelectric materials [7]. The electric current free energy is obtainable by integration of current density distribution across the materials as follows [8]

1.3 $$G_{e}=-\frac{1}{8\pi }\int \int \frac{\mu (r^{\prime })j(r)\cdot j(r^{\prime })}{|r-r^{\prime }|}\;\text{d}r\;\text{d}r^{\prime },$$

where ***j***(*r*) is the electric current density at a space position *r*. The equation has been implemented successfully in the prediction of many current-driven phenomena. For example, Dolinsky & Elperin applied the equation to calculate the explosion of current-carrying wire to form nanoscale particles [9]. The evolution of surface profile influences the electric current distribution in the bulk materials. This changes the electric current free energy sequence between various surface geometries and hence alters the free energy asymmetry Δ in equation (1.1). The effective relaxation time *τ* for the surface evolution is hence affected by the electric current. For a solidification in which relaxation time matters, application of electric current has the potential to generate the desired effect to improve the surface properties.

Application of pulsating solidification to oxides is driven by engineering requirements from several industrial companies via a collaborative project. The surface of a solidified oxide mixture is required to be as smooth as possible in order to use it to manufacture new products. The solidified oxide mixture is very brittle and unable to be polished by the traditional surface processing methods such as rolling, sanding, laser polishing, abrasive fluid jet, magnetic abrasive machining and chemical treatment. Its surface is usually very rough. The molten oxide mixture is electric conductive (via the moving ionized atoms [10]) in liquid and semi-solid states, which provides an ideal case to validate the proposed theory on pulsating solidification. The experiments, including materials, methods and results, are reported in §2. The experimental observations have been implemented to an *in situ* numerical calculation. The procedures and discussions are shown in §3. This is followed by the conclusions in §4.

## 2. Experiments

**Figure 1.**
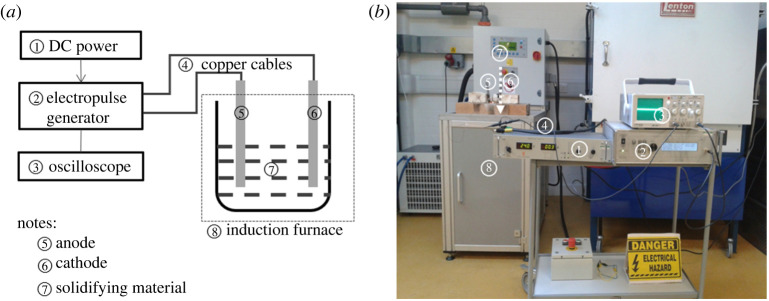
(*a*) Schematic diagram for experimental set-up, (*b*) photograph of experimental installation. (Online version in colour.)

A mixture of oxide powders with chemical constituents in wt.% at 32.9 CaO, 23.5 SiO_2_, 7.64 Na_2_O, 7.51 Al_2_O_3_, 6.56 F, 5.90 MnO, 0.79 MgO, 0.31 TiO_2_ and 14.80% C + CO_2_ was implemented to make the samples. The powder was provided by Sandvik AB, Sweden. The schematic diagram for experimental set-up is illustrated in figure 1*a*. The actual experimental installation in the laboratory is shown in figure 1*b*. The oxide powders were put into a graphite crucible and melted by induction heating at ambient environment before turning off induction for pulsating solidification. The sample's melting temperature is 1423 K. Two preheated iron bars were inserted to the molten oxides to act as electrodes.

**Figure 2.**
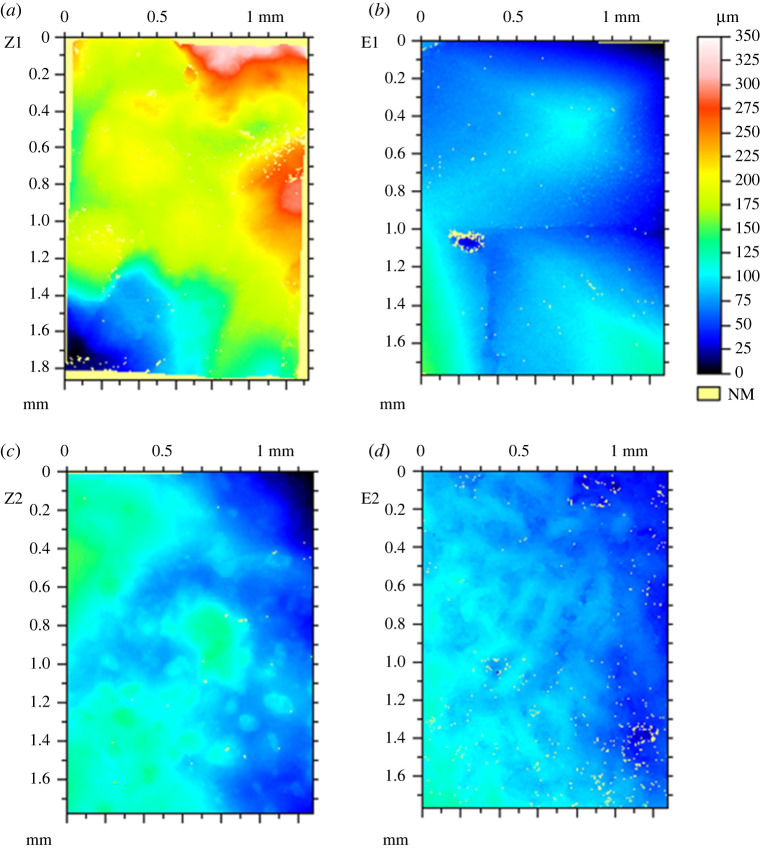
Two-dimensional surface profiles were obtained using a Leica DCM-3D Profiler. The left column images, (*a*) and (*c*), are from the samples without pulsating. The right column images, (*b*) and (*d*), are from pulsated samples. The upper row images are taken from a position (position 1) nearer to the electrodes than that of the bottom row images (position 2) and hence solidified quicker. (Online version in colour.)

In pulsating solidification, the electrodes were connected to an electric current pulses generator to load 50 A peak current, 50 µs pulse duration and 100 Hz pulse frequency. The pulses are in square shape. The electric current was observed to decrease monotonically during cooling and vanished at around 700 K. During the solidification, the system was cooled down in open air at ambient conditions. According to thermocouple measurement, the temperature was dropped from 1473 K to 573 K in 1600 s, which gives an average cooling rate of 0.56 K s^−1^. The reference samples were solidified at the same conditions as those of pulsated samples except without the loading of electric current pulses to the inserted electrodes. The surface geometries of both pulsated and reference samples were characterized using a Leica DCM-3D Profiler. Each sample was examined at 12 different positions across the free surface. Only two positions are discussed in the present work because they represent the two extreme conditions. The other 10 positions are between them and their roughness changes are among the discussed cases. Figure 2 presents the surface profiles of samples without and with pulsating at two characteristic positions. They are (a) position 1 without pulsating (labelled as Z1), (b) position 1 with pulsating (E1), (c) position 2 without pulsating (Z2) and (d) position 2 with pulsating (E2). The colour spectrum from blue (dark grey in black-white pattern) to red (light grey in black-white pattern) represents the height of the surface from the measured minimum to the measured maximum. The unmeasured points are given the average measured value and hence represented by yellow colour.

In comparison with position 2, position 1 is closer to the electrodes and hence solidifies quicker. This is due to the lower temperature in electrodes than in molten oxides. Figure 2 shows clearly that the pulsed samples have less amplitude of surface fluctuation than in the corresponding samples without pulsating. The surface roughness represents a probability distribution of surface heights around its average surface height [11], which is characterized using the following expression [12]

2.1 $$R_{a}=\frac{1}{l_{r}}\int_{0}^{l_{r}}|z(x)-\bar{z}|\;\text{d}x,$$

where *l_r_* is the integration length, *z*(*x*) the height of surface and $\bar{z}$ the average height in the integrated surface profile. The roughness for the images in figure 2 has been calculated. The results are listed in table 1. It shows that pulsating reduces surface roughness by over 50% for both the quenching affected (position 1, adjacent to electrodes) and less affected areas (position 2, away from the electrodes). The insertion of electrodes disturbs surface geometry, and the quicker solidification reduces relaxation time. The roughness at position 1 is hence much larger than that of position 2.

**Table 1.** Surface roughness (*R*_a_-value) and pulse-induced roughness reduction.

position	*R*_a_ of reference sample (µm)	*R*_a_ of pulsated sample (µm)	reduction (%)
1	42.61	17.39	59.19
2	21.18	7.90	62.70

## 3. Discussion

To quantitatively investigate the influence of electromagnetic field on the surface evolution, both the surface energy and electric current free energy in the samples without and with pulsating are required to calculate the effective structural relaxation time. This demands all surface geometric information. However, experimental characterization of surface profile reveals thousands of surface points which are unmeasurable. Those points are from the areas with very steep incline, which stop any significant light returning to the camera. For example, the total number of points in each measurement in figure 2 is 1065 × 768 = 817 920 points. The unmeasured number of points in figure 2 is found to be at (a) 26 985, (b) 18 026, (c) 5670 and (d) 10 212, respectively. Those unmeasured points are shown as yellow spots in figure 2 and labelled as NM in the legends. It is also noticed that the unmeasured points are frequently segregated, which makes the straightforward mathematical interpolation methods powerless.

### (a) Interpolation of the unmeasured surface points by artificial neural network

A computational code package based on the supervised learning using multi-layer neural networks has been developed to interpolate the unmeasured surface height from the measured points. The methodology for multi-layer neural network was described by MacKay [13]. Its application to materials science was described constructively by Bhadeshia [14]. The neural network mapping function implemented in the present work is defined as

3.1*a* $$a_{j}^{\left(i\right)}=\overset{N^{\left(i-1\right)}}{\underset{l=1}{\sum }}w_{jl}^{\left(i\right)}h_{l}^{\left(i-1\right)}+b_{j}^{\left(i\right)}$$

and

3.2*b* $$h_{j}^{\left(i\right)}=f^{\left(i\right)}\left(a_{j}^{\left(i\right)}\right)$$

where $a_{j}^{\left(i\right)}$ is called the activation of *j-*th unit in *i-*th layer network. *N*^(*i*−1)^ is the total number of units in (*i* − 1)-th network layer. $w_{jl}^{\left(i\right)}$ is the weight factor between *j-*th unit in *i-*th layer and *l-*th unit in (*i* − 1)-th layer. $h_{j}^{\left(i\right)}$ and $b_{j}^{\left(i\right)}$ are the output and bias of *j-*th unit in *i-*th layer, respectively. *f*^(*i*)^ is the activation function at *i-*th layer. $h_{j}^{\left(0\right)}$ is in fact the *j-*th input parameter. *N*^(0)^ is the total number of input parameters. For a network containing *m* layers, $h_{j}^{\left(m\right)}$ is the *j-*th final output parameter. Two types of activation function have been implemented to train the networks in this calculation, namely the linear (*f*(*a*) = *a*) and sigmoid (*f*(*a*) = tanh(*a*)) functions. The target function is defined as follows.

3.2 $$T(w)=\frac{1}{2}\beta D+\frac{1}{2}\alpha \underset{j,l,i}{\sum }{\left[w_{jl}^{\left(i\right)}\right]}^{2},$$

where *β* is a pre-factor coefficient. *D* is the total difference between the targeted values and neural network calculated values

3.3 $$D=\frac{1}{n}\underset{n}{\sum }\underset{j}{\sum }{\left(t_{j}^{\left(n\right)}-h_{j}^{\left(m)(n\right)}\right)}^{2},$$

$t_{j}^{\left(n\right)}$ is the *j-*th targeted parameter at *n*th training dataset. *α* is called the weight decay rate. The second term in equation (3.2) is called weight decay regularizer, which helps to decrease the tendency of a model to overfit noise in the training data [15]. The artificial machine learning is to perform a gradient descent minimization of the target function as

3.4 $$\Delta w_{jl}^{\left(i\right)}=-\eta \partial T\left(w_{jl}^{\left(i\right)}\right)/\partial w_{jl}^{\left(i\right)}$$

where *η* is called learning rate. Each weight factor is modified step-by-step according to equation (3.4) to minimize the difference between the targeted values and calculated values using the backpropagation algorithm. The code was validated against several analytical functions including parabolic, double-well and sinusoidal artificial distributions. Convergency was achieved quickly and the data fitting between the calculation and analytical results was found to have high accuracy. For further validation, the code package was implemented to calculate several artificial learning cases described by MacKay [13]. The same results were obtained.

The measured data contains between 791 025 sets of data in figure 2*a* to 812 250 sets of data in figure 2*c*. They are implemented to train and validate the parameters in the neural network. A high-quality random number generator was coded according to the mathematical theory developed by Marsaglia *et al.* [16]. This has been implemented to randomly select 90% measured data to train the neural network and to leave the remaining 10% data for validation. The purpose of training the neural network is to find out the weight factors and bias values in equation (3.1).

**Figure 3.**
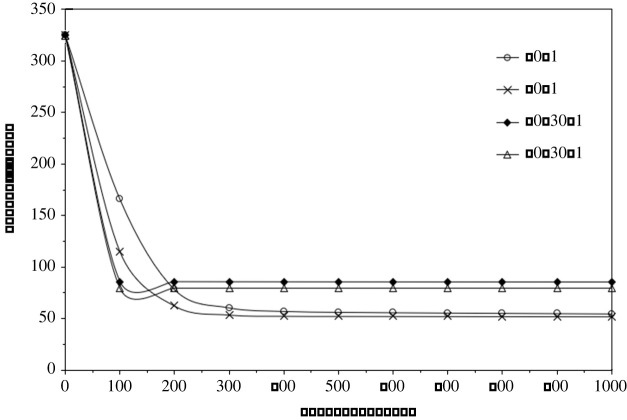
The evolution of total difference (*D*) versus iteration steps in validation. The legend label 70-1 means a two-layer neural network with a hidden layer containing 70 units and output layer having 1 unit. 40-30-1 means a three-layer neural network with the first hidden layer containing 40 units, the second hidden layer with 30 units and output layer having 1 unit.

Due to high fluctuation of the surface profile, using one neural network to compute the whole surface can cause considerable discrepancy. To overcome this problem, the 1065 × 768 lattice is divided into 120 units with each unit containing a 71 × 96 lattice. This type of grouping method has been successfully reported in the calculation of materials properties by Haraguchi *et al.* [17]. All the input and target datasets in each unit are normalized to between 0 and 1. The normalized values are calculated by multi-layer neural network to find the desirable parameters of weight factors and bias. These parameters are initially assigned to a random value between 0 and 1. An artificial learning by backpropagation algorithm is implemented to optimize the parameters. The optimized ones are then implemented to calculate the unmeasured values. Using *β* = 0.5, *α* = 0.001 and *η* = 0.05, numerical calculation shows that a modified learning rate after 500 times iteration to *η*/(0.1*t*) is better than that of the fixed one, where *t* is the iteration time step. In order to find out the most suitable number of network layers and the number of units in each layer, many trials have been performed before the final optimization. Figure 3 illustrates some cases for the convergence behaviour of *D* versus iteration steps. The plotted cases have sigmoid activation function and each unit with a bias. Linear activation function was also tested for the output layer but shown to be worse than that using the sigmoid one. Three-layer neural networks use more computing time without producing better results than two-layer networks at the same number of total units. It is noticed from the calculation that two-layer neural networks with a hidden layer containing 70 units perform nicely. The iteration time of 1000 is sufficient for the desirable accuracy although 150 000 time steps have been examined.

**Figure 4.**
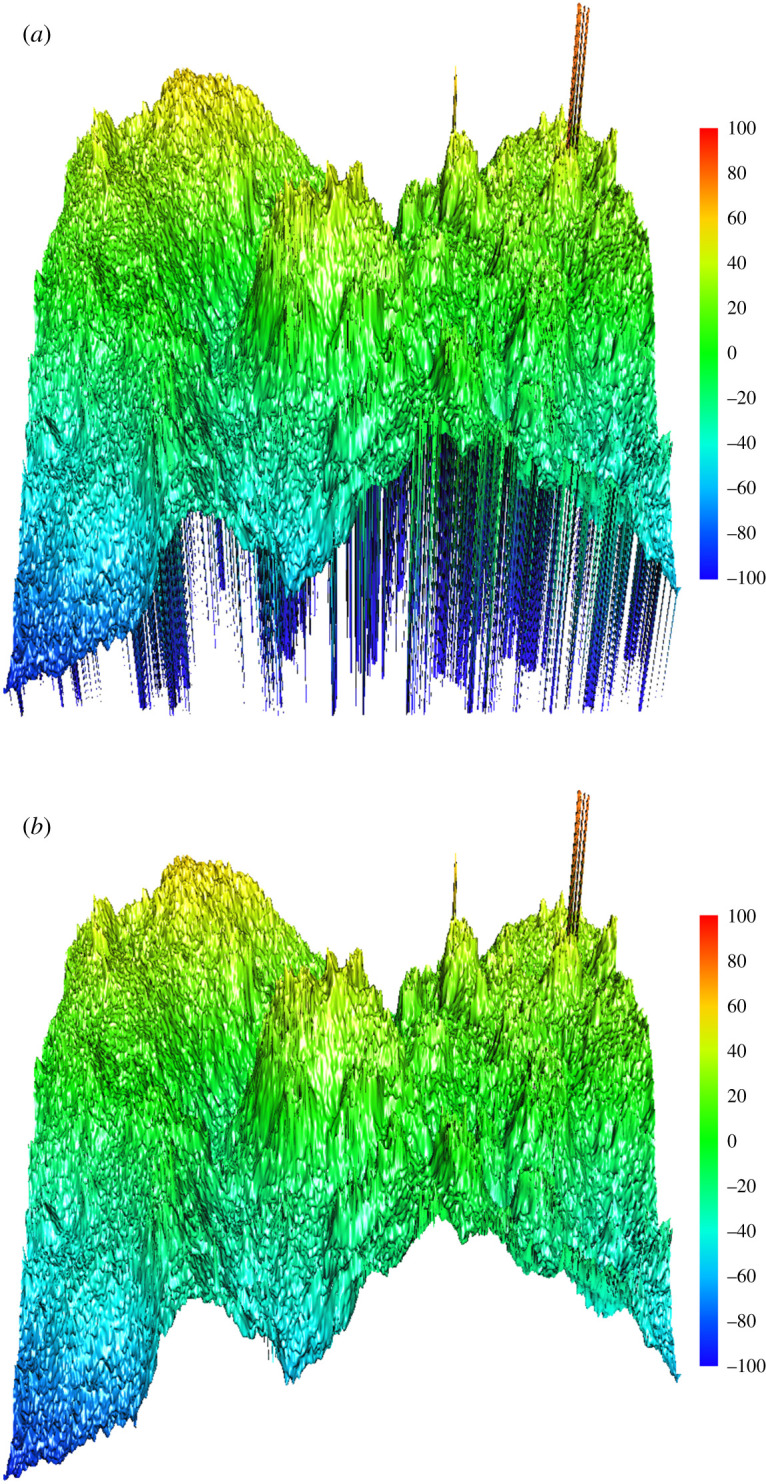
The surface profiles for Z2 with (*a*) all the unmeasured points assigned to −100 µm, and (*b*) the unmeasured data replaced by neural network interpolated values. (Online version in colour.)

In figure 3, 70-30-1 represents three-layer neural networks with the first layer consisting of 70 units, the second layer of 30 units and third layer of 1 unit, while 60-1 indicates two-layer neural network with the first layer consisting of 60 units and second of 1 unit. Figure 4 plots the Z2 surface before and after the neural network interpolation using MatVisual software, where figure 4*a* assigned all the unmeasured data to −100 µm after consideration of the measured minimum value of −91.4 µm. Figure 4*b* has all the unmeasured data replaced with the interpolated values from neural network calculations. The validation reveals a reduction of 83.9% total difference after 1000 iteration times. It achieves 90.1% reduction after 400 000 times. The neural network calculation modifies slightly the surface roughness and average height, as is shown in table 2.

**Table 2.** The corrected surface roughness and average height after interpolation.

sample	parameter	before (μm)	after (μm)
E1	roughness	17.3869	17.2986
Z1		42.6083	42.8881
E2		7.90474	7.93931
Z2		21.1794	21.1692
E1	average height	4.78 × 10^−7^	−0.032
Z1		3.18 × 10^−6^	0.157
E2		3.97 × 10^−7^	−0.013
Z2		2.90 × 10^−7^	−0.034

### (b) Computation of electric current free energy using the realistic surface profile

**Figure 5.**
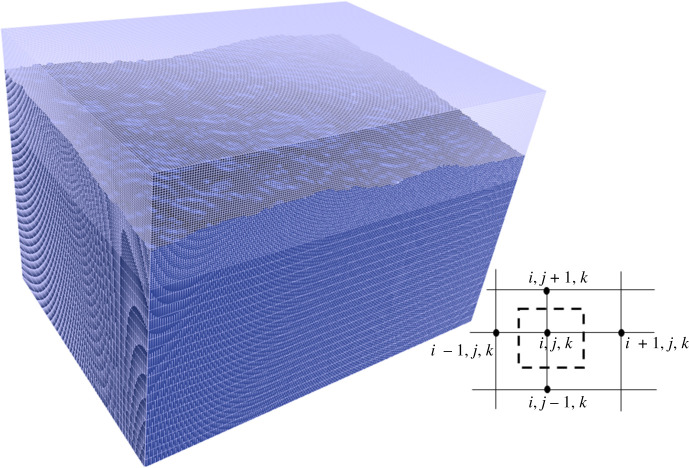
The adaptive lattice and the system for the Z2 surface. The embedded box in the right bottom corner formed by dashed lines shows the grid volume. (Online version in colour.)

The measured surface profiles and the interpolated data for the unmeasured sites are implemented to calculate the electric current free energy. In three-dimensional computation, however, a logic frame containing a 1065 × 768 lattice in x–y plane is computationally over-demanding for a workstation. It therefore skips four grids along the x and y axes after taking each grid's coordinates, respectively. The choice of the number of skipped grids is dependent on the computational power and desirable accuracy. A smaller number means more computing time and a larger number causes low accuracy. This reduces the number of grids in the plane to 213 × 154. The corresponding dimensions are 1.7596 mm × 1.2699 mm. The lattice distance in the plane is 8.3 µm. Along the z-direction, the material's average height is defined to be 1 mm with the top surface at a fluctuation amplitude no more than 0.2 mm. This is satisfied as the largest surface fluctuation amplitude among the four cases in figure 2 is between -0.184 mm and 0.181 mm in Z1. The other five surfaces are assumed to be smooth. The computational logic frame therefore has a height of 1.2 mm. Two grid distances are implemented along the z-direction, i. e. 20 grids at 40 µm distance from *z* = 0 to *z* = 0.8 mm and 51 grids at 8.0 µm distance from *z* = 0.8 mm to *z* = 1.2 mm. In space initialization, all the grids with *z*(*x*, *y*) ≤ 1.0 + *z_m_*(*x*, *y*) are assigned to a phase field order parameter 1 to represent oxide material and the other lattices with *z*(*x*, *y*) > 1.0 + *z_m_*(*x*, *y*) are assigned to a phase field order parameter 0 to represent atmosphere, where *z_m_*(*x*, *y*) is either the measured or interpolated surface profile data. The three-dimensional computational logic frame consists of 213 × 154 × 71 nodes, as is shown in figure 5 for the Z2 surface profile.

The next problem is to choose the electrical conductivity of the sample. There are two considerations. The first is that the observed peak electric current in experiments is around 50 A. The second is that the integration in equation (1.3) goes throughout the space with non-zero current density. To capture those two features in the calculation area, an electrical conductivity of the liquid materials is implemented at 1.0 × 10^7^ S m^−1^, which is in the same order as that of steel but higher than that in the molten oxides. The assumption is reasonable because the electric current free energy is directly related to electric current rather than resistance. The atmosphere is not conductive, which is approximated using a very low electrical conductivity value at 1.0 S m^−1^ (i.e. seven orders of magnitude lower than that in liquid). The material is subjected to 1 V electric potential along the longest dimension. The magnetic permeability for both the oxide materials and atmosphere equals the vacuum permeability *μ*_0_ = 1.25663706 × 10^−6^ H m^−1^.

In discrete space, equation (1.3) can be discretized to the following format according to Qin & Bhowmik [8]

3.5 $$G_{e}=-\frac{1}{8\pi }\underset{ijk}{\sum }\underset{i^{\prime }j^{\prime }k^{\prime }}{\sum }\frac{\mu (i,j,k)j(i,j,k)\cdot j(i^{\prime },j^{\prime },k^{\prime })V(i,j,k)V(i^{\prime },j^{\prime },k^{\prime })}{|r(i,j,k)-r^{\prime }(i^{\prime },j^{\prime },k^{\prime })|},$$

where (*i*, *j*, *k*) and (*i′*, *j′*, *k′*) represent two nodes in the computational logic frame. *V*(*i*, *j*, *k*) is the volume element of node (*i*, *j*, *k*). The volume element of a grid is an enclosure formed by six perpendicular bisector planes between the node and its six neighbouring nodes, as is shown in the embedded diagram in figure 5. The electric current density between two neighbouring nodes *α* and *β* is obtained by Ohm's law as

3.6*a* $$j_{\alpha \beta }=(U_{\alpha }-U_{\beta })\cdot \frac{\sigma_{\alpha \beta }{\hat{e}}_{\alpha \beta }}{S_{\alpha \beta }},$$

where *U_α_* is the electric potential at grit *α*, σ*_αβ_* is the electrical conductance between two grids *α* and *β*, *S_αβ_* is the cross-section area between *α* and *β* and ${\hat{e}}_{\alpha \beta }$ the normal vector of the cross-section plane. To calculate the electric current according to equation (3.6), the distribution of electric potential across the computational system needs to be obtained first. To this end, the relaxation method has been implemented. The initial conditions include the values of applied electrical potential at two electrodes and an assumed gradient distribution of electric potential from anode to cathode. The relaxation method uses time iteration to obtain a static distribution to satisfy the defined boundary conditions. According to Kirchhoff's laws, the iteration of the electric potential is undated according to

**Figure 6.**
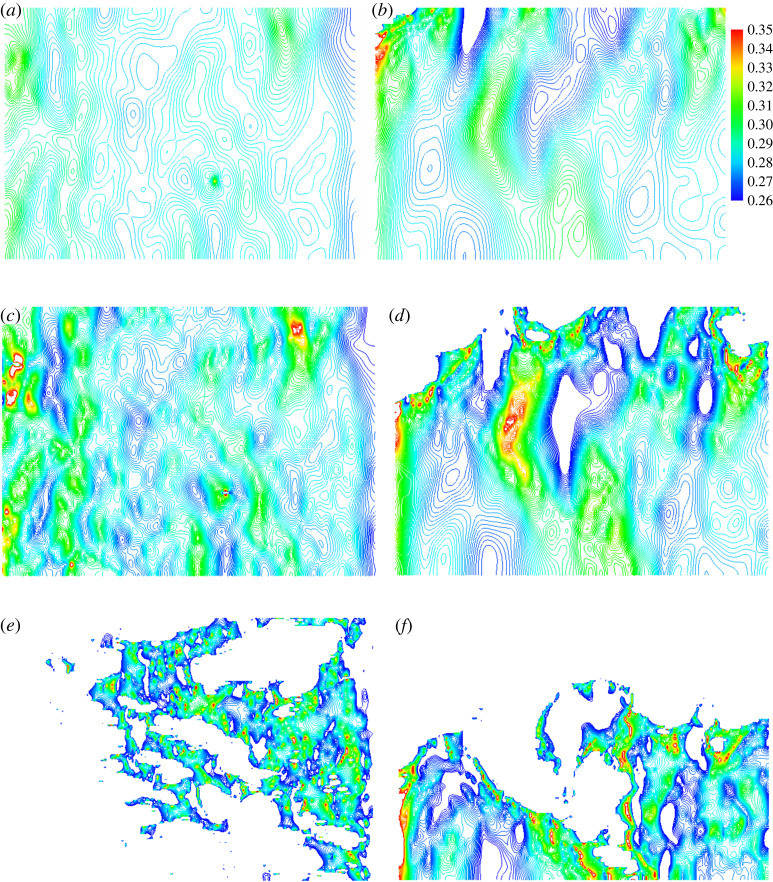
The current distribution for Z2 (left column) and E2 (right column), where (*a*) and (*d*) at *z *= 35, (*b*) and (*e*) at *z *= 40, and (*c*) and (*f*) at *z *= 45. (Online version in colour.)

3.6*b* $$U_{\alpha }(t+1)=\frac{\sum_{\beta }U_{\beta }(t)\sigma_{\alpha \beta }}{\sum_{\beta }\sigma_{\alpha \beta }},$$

where *t* is the iteration step. The summation goes over all the nearest neighbours *β* of a considered grid *α*. The boundary conditions include the fixed electrical potential at left and right surfaces and zero flux in all the lateral surfaces. After each time iteration, the total change of electric potential distribution ($\sum_{\alpha }|U_{\alpha }(t+1)-U_{\alpha }(t)|$) is obtained to decide whether the relaxation has achieved a satisfactory accuracy. In the present calculations, the time iteration has been defined to 150 000 time steps. This allows the change of electric potential across all the grids between two adjacent iteration steps less than 10^−7^ volts. Figure 6 presents the contour of electric current density distributions for Z2 (left column) and E2 (right column), where (a) and (d) are for the section at z = 35, (b) and (e) at z = 40, and (c) and (f) at z = 44 grid layers in the z direction. The unit in figure 6 is 10^10^ A m^−^^2^.

According to analytical calculation, the application of 1 V electric potential to a rectangular parallel piped material with electrical conductivity 1.0 × 10^7^ S m^−1^ and dimensions at length 1.7596 mm, width 1.2699 mm and height 1 mm generates a uniform electric current density of 5.683 × 10^9^ A m^−2^. Figure 6 shows that the surface profile affects the distribution of the electric current significantly. The material with a less rough surface has more uniform current distribution. The different current density distributions cause different electric current free energy. The numerical integration results for the electric current free energies are listed in table 3. The results show that E1 has lower electric current free energy than Z1 and E2 has lower electric current free energy than Z2. The reduction of surface roughness at the same point is along the trend to reduce the electric current free energy. MatVisual software has an embedded functionality to calculate the surface area. The results are obtained and presented in table 3. The surface area from Z1 to E1 is reduced, which is easy to understand as surface relaxation tends to minimize the surface energy. However, the surface area has been increased from Z2 to E2, which is contradictory to the general knowledge in surface energy-driven surface evolution. This is attributed to the effect of pulsating processing to be discussed in the following.

**Table 3.** The electric current free energy and surface area from numerical calculations.

sample	electric current free energy (10^−3^ J)	surface area (10^−6^ m^2^)
Z1	−2.65592	2.6196
E1	−2.69339	2.5481
Z2	−2.69538	2.4960
E2	−2.69824	2.5353

### (c) The mechanisms

The surface tension of the molten oxide mixture is around 0.4 N/m according to the assessment by Mills & Keene [18]. The change of surface energy from Z1 to E1 is −2.86 × 10^−8^ J and from Z2 to E2 is 1.57 × 10^−8^ J. For the electric current free energy, the change from Z1 to E1 is −3.747 × 10^−5^ J and from Z2 to E2 is −2.86 × 10^−6^ J. The reduction of electric current free energy is significantly greater than the surface energy change. Without electric current, surface evolution from Z2 to E2 is impossible due to its increment of surface area. With electric current, this is possible because the increment in surface energy can be compensated by a larger reduction of electric current free energy. This calculation has provided further evidence that pulsating processing can help to break the conventional restriction and generate new phenomena. Furthermore, the driving force from the electric current is over 100 times larger than that of the surface energy. The relaxation time, according to equation (1.1), is hence reduced significantly. The structural relaxation is accelerated vastly.

An issue in need of clarification is that the surface area in E2 is larger than that in Z2 but the roughness in E2 is smaller than Z2. This is possible and can be illustrated using an example. For a surface profile described by sinusoidal equation *z*(*x*, *y*) = *A*sin(*ωx*), its roughness is proportional to the amplitude *A* but has nothing to do with the frequency *ω* [3]. The surface area, however, is proportional to both *A* and *ω*. A smaller *A* but larger *ω* would enable the surface roughness to be smaller but surface area to be larger, which is similar to the situation between E2 and Z2. A surface is described by both the roughness and texture (frequency of surface wavelets) in metrology [19] but only described by roughness in noise theory [20].

Another issue in the present calculation is that the electric current free energy is neither always a monotonic function of roughness nor surface area. An exception is shown in table 3 between E1 and Z2. Although electric current shows a significant effect to reduce surface roughness, some strong local fluctuation can affect electric current distribution in a much wider length scale to affect drastically the electric current free energy, while the local fluctuation only affects the local contribution to the roughness and surface area. An extreme example is that a local fluctuation can cut the conductive materials thoroughly and stop electric current completely. However, such an extreme situation does not affect the general conclusion of electropulse-induced roughness deduction.

The electric current density implemented in the present work is around 5.683 × 10^9^ A m^−2^. In the previous works reported in the literature, an electric current density at 9.61 × 10^9^ A m^−2^ was implemented in the fabrication of nanostructured pearlitic steel [21] and 1.018 × 10^7^ A m^−2^ was used in the microstructural modification of low carbon autonomous steels [22]. All these indicate a strong thermodynamic effect from the electromagnetic field. It is possible that the reported experiments in electropulsing-assisted ultrasonic surface rolling process [1] and alternating magnetic field treatment induced roughness modification [2] consist of the same mechanisms. However, further investigation is required for those experiments as the former combines the electropulse with ultrasonic processing and the latter contains a second-order phase transition. Regarding the side effects, Ohm heat was minimized by the implementation of short pulse duration (50 µs) and low pulse frequency (100 Hz). As is well-known, the application of an alternating electric current within a conductor causes the current density to be largest near the surface of the conductor. It is called skin effect and the skin depth is inversely proportional to the square-root of the frequency. The skin effect is favourable to the surface treatment and the skin depth is larger than the considered materials thickness.

According to equation (1.3), the electric current free energy is proportional to the average electric current density square. The latter is proportional to the applied electrical potential and the materials electrical conductivity. For the processing of different materials with different electrical properties, different electric potential can be implemented to ensure the impact of the pulsating processing.

**Figure 7.**
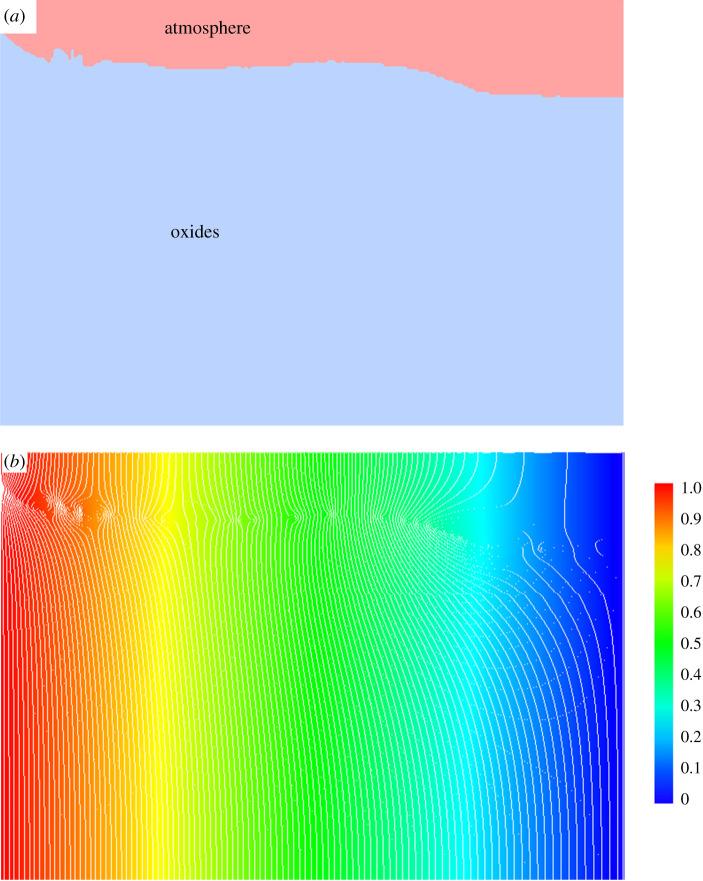
A slice at *y *= 74 grid slice for (*a*) phase field for E1 and (*b*) contour of electrical potential for Z1, where the legend in (*b*) is represented the electric potential. (Online version in colour.)

Another observation is the distortion of electrical potential gradient across the interface. The surface can be considered as an interface between oxides and atmosphere, as is shown in figure 7*a* for E1 at y = 74 grid layer. A high-resolution visual analysis of contour lines indicates a slightly distorted electrical potential gradient, as is denoted by the arrows in figure 7*b*, the image shows some vortexes around the interface position. The distortion of electric current potential, although very weak, indicates the effects of electric field on the interface. It is not known whether this contributes to the interface energy. However, such an assumption was made two decades ago by Barnak *et al*. [23] in alloys solidification but has never been approved either in theory or experimental measurement. On the other side, the effect of electric field on the interface evolution of dielectric materials has made significant progress. For example, Brown *et al.* reported using the effect to synthesize optical components [24]. The mechanisms, however, are different as the latter is from heterogeneous polarization around the interface.

## 4. Conclusion

(i) Application of electric current pulses to the solidifying conductive materials promotes the relaxation of surface fluctuation. The experiments show a greater than 50% reduction of surface roughness in the pulsated oxide samples.
(ii) The electric current free energy, according to calculation, imposes a thermodynamic driving force for surface relaxation. It can be 100 time stronger than the surface energy.
(iii) The artificial multi-layer neural network has been implemented to interpolate the unmeasurable data.
(iv) A numerical calculation based on realistic surface profile has been performed to calculate the electric current free energy. The numerical results are in agreement with the experimental observations.
(v) The distortion of electric field around the interface has been noticed. It is possible that the interface energy is affected by the distortion.

## Data Availability

Availability of data and material (data transparency). The datasets generated during and/or analysed during the current study are available from https://ordo.open.ac.uk/articles/dataset/Supplementary-materials-for-RSPA-2021-0726/19487840/1. Code availability (software application or custom code). Figures 4–7 are plotted using MatVisual commercial software.
